# Community environmental satisfaction: its forms and impact on migrants’ happiness in urban China

**DOI:** 10.1186/s12955-018-1061-1

**Published:** 2018-12-18

**Authors:** Sainan Lin, Yinxuan Huang

**Affiliations:** 1 School of Urban Design, Wuhan University, Wuhan, China; 2 Manchester Urban Institute, School of Environment, Education, and Development, The University of Manchester, 1.34 Humanities Bridgeford Street Building, Oxford Road, M13 9PL, Manchester, UK

**Keywords:** Subjective well-being, Community environmental satisfaction, Migrants, Cohort difference, Urban China

## Abstract

**Background:**

The great number of internal migrants has become an important part of China’s urban population. Improving migrants’ well-being is emerging as a key to the state policy emphasized in China’s New-type Urbanization Plan. Previous studies on subjective well-being (SWB) have primarily focused on the impacts of objective measures of community environment and consider migrants as a homogeneous group. This study extends the literature by exploring the impacts of perceived community environment on migrants’ SWB and incorporating cohort differences in the analysis.

**Methods:**

We use the 2015 national scale data—China Household Financial Survey (CHFS) data—to analyse the different forms of community environmental satisfaction and their impacts on migrants’ subjective well-being. A total of 12,607 migrants were sampled from 29 of mainland China’s 31 provinces. Latent class analysis is applied to explore the potential forms of community environmental satisfaction; multinomial and ordinal logistic regression models are constructed to examine the sociodemographic characteristics of different forms of community environmental satisfaction and the association between community environmental satisfaction and subjective well-being among migrant cohorts in urban China.

**Results:**

Latent class analysis defines four distinctive latent classes, which mirror four different domains of migrants’ perception of their local environments. They are called ‘unsatisfying local environment’, ‘satisfying social environment’, ‘satisfying physical environment’, and ‘satisfying social life’. Results from the multinomial logistic regression model reveals that the four forms of community environmental satisfaction are underpinned by distinct sociodemographic characteristics. Results from a series of ordinal logistic regression models show that different forms of community environmental satisfaction, in particular satisfaction with the physical environment and with social life, are positively associated with migrants’ happiness. The model results also suggest that cohort differences do exist among migrants. The positive effect of a satisfying physical environment on happiness tends to be greater in younger cohorts, while the positive effect of a satisfying social life on SWB is more observable in older cohorts.

**Conclusion:**

Satisfaction with community environment has a salient impact on urban Chinese migrants’ happiness. For their SWB, improving migrants’ physical living environments and social lives is relatively more important than social environment, which in a way mirrors migrants’ current status with its deficiencies in terms of a comfortable living environment and social life. Moreover, there exist cohort differences that should be considered when making policies to enhance migrants’ subjective well-being.

## Background

The transformation of post-reform urban China is marked by the unprecedented scale of internal migration. According to the National Bureau of Statistics of China, there were 245 million migrants in Chinese cities as of 2016, comprising a significant proportion of the urban population. Improving migrants’ well-being is emerging as a key to state policy as emphasized in China’s New-type Urbanization Plan. Extant studies investigating city-dwelling migrants’ well-being [1–4] have found that the household registration (*hukou*) system creates remarkable disparities in well-being between migrants and urban residents. Migrants, often regarded as a socio-economically and institutionally disadvantaged group, have limited access to stable jobs, housing and social welfare and therefore experience a lower-than-standard quality of life [5]. However, until recently, only the objective well-being of migrants has been examined; more attention is now being paid to their subjective well-being (SWB) [5–8]. SWB, also referred to as ‘life satisfaction’ or ‘happiness’, is defined by Diener as “people’s evaluations of their life as a whole or of its various domains” [9]. The very few studies on migrants’ SWB in China have revealed that migrants have lower levels of SWB than both urban residents and rural residents [7, 10]. Migrants’ high aspirations in achievement or influenced by their new reference groups result in their unhappiness in the city [10]. Moreover, many research has shown that higher levels of SWB has objective benefits such as increased productivity, creativity, higher income, physical health and sociability [11] (See De Neve et al., 2013 for an overview of the objective benefits of subjective well-being). Vice versa, the lower levels of SWB among migrants may result in negative outcomes. As such, understanding the determinants underlying migrants’ SWB is important for China’s goal of ‘common prosperity’ and ‘people-centred’ urbanization.

Existing studies have identified two major categories of determinants associated with SWB. The first category is personal demographic and socioeconomic status (SES), including but not limited to age, education, occupation, income and health [6, 12, 13]. For example, income as a factor influencing SWB has been widely explored and is generally positively associated with SWB [6, 14, 15]. The second category is residential environment [16, 17]. The literature on this component of SWB has burgeoned over the past decade. Recent research has shown that a person’s SWB is greatly influenced by both physical and social environment [18]. With regard to the effects of physical environment, Cao discovered that high population density and poor neighbourhood street connectivity are detrimental to residents’ SWB [19]. Although there is no consensus about the definition of social environment, the social environment in which individuals live influences behaviour by “shaping norms, enforcing patterns of social control, providing or not providing environmental opportunities to engage in particular behaviours, reducing or producing stress, and placing constraints on individual choice” [20, 21]. Li and Rose found that individuals’ social connectedness, reflected in issues such as social capital, social exclusion, and identity, has been shown to have significant impact on SWB in both China and Western societies [22].

From the above literature review, we find that the current scholarly understanding of the effects of residential environment on SWB is principally based on objective residential environment and that little attention has been paid to subjective or perceived residential environment. Gidlöf-Gunnarsson and Öhrström, however, are an exception; they found that perceived availability to nearby green spaces positively affects residents’ SWB [23]. As Logan and Collver posited, “residents’ perceptions of what their community and other communities are like are as important to urban theory as the information on objective characteristics on which most urban research is based” (1983: 432) [24]. As such, perceptions of community environment or community environment satisfaction are well worth exploring when studying the effects of residential environment on residents’ SWB.

After the reform in the end of 1970s, China gradually relaxed its migration policies, which allow people to move freely from rural to urban areas. As such, the pre-70s generation, who were young adults at that point, has become the first generation of rural to urban migrants. Entering the 21 century, migrants who were born after 1980s have become the major group; they are known as the “new generation” [25]. The first and new generation of migrants, who grew up in total different social-political environment—one in the era of planned economy and the other in the reform era, may differ in terms of livelihood, socioeconomic integration, and personal pursuits [4, 26]. For example, He and Wang revealed that the new generation migrant has comparatively higher level of SWB than the first generation, due to their higher educational attainment, more colorful recreational activities and lower family burdens [4]. However, the current research failed to address the further differences among different cohorts. Particularly after 2010, with the growing number of migrants who were born after 1990s, China’s internal migrants has become an even more heterogeneous group. Research has found that the migrants who were born after 1990s have the lowest integration level than their previous generations [27]. As such, it is important and urgent to address the cohort differences by subdividing them to Pre-70s, Post-70s, Post-80s, and Post-90s, rather than simply comparing first and new generations.

This article aims to fill the abovementioned knowledge gaps by using data from a large-scale national survey—the 2015 China Household Financial Survey (CHFS)—to examine the impacts of community environmental satisfaction on migrants’ SWB. In particular, this study seeks answers to two research questions: (1) What are the forms of community environmental satisfaction and how do they associate with migrants’ SWB? (2) Are there cohort differences in terms of SWB and, if so, what are their patterns?

## Methods

### Data

We use data from the 2015 China Household Financial Survey (CHFS), which is the third wave of the panel study. CHFS is managed by Southwestern University of Finance and Economics and aims to gather evidence on a wide range of financial and socioeconomic activities in Chinese society. Covering 29 of China’s 31 mainland provinces, this national representative survey was able to draw information from 100,000 individual respondents in 40,000 households in its 2015 wave. CHFS uses a stratified three-stage probability proportion to size (PPS) random sample design. The three stages of sampling are counties, residential communities from the counties, and extracted households from the neighbourhoods and village committees. PPS was used for all three stages, which were also weighted by population size. The attention devoted to migrants in the Chinese city motivated us to restrict our study population to adult (aged 18 or above) migrant respondents in the urban sample of the CHFS. Specifically, migrants were defined as individuals whose *hukou* were not registered in the host city, but who had resided in the city for at least six months at the time of the survey interview. Consequently, the final sample size for the empirical analysis was 12,607. CHFS data collectors provided sampling weights for selection biases, which are used in all analyses reported in this paper.

### Independent variables

In order to explore the cohort differences, we first classified the study population into four groups according to respondent birth cohort in order to examine the differences across generations of migrants. The classification is exhibited in Table 1. Individuals who were born before 1970 were called ‘Pre-70s’, while those who born between 1970 and 1979 were called ‘Post-70s’. ‘Post-80s’ and ‘Post-90s’ refer to adults who were born between 1980 and 1989 and between 1990 and 1996, respectively. This classification has been widely used in sociological studies and news commentaries on generational issues in China [28, 29]. Table 1 shows that nearly 50% of the CHFS urban migrant sample consisted of individuals born prior to 1970. The shares of Post-70s, Post-80s, and Post-90s are 18, 22, and 14%, respectively.

**Table 1:** Sample Characteristics

	Weighted % or Weighted mean (SD)				
	All	Pre-70s	Post-70s	Post-80s	Post-90s
Cohort					
Pre-70s	46%				
Post-70s	18%				
Post-80s	22%				
Post-90s	14%				
Hukou					
Non-rural	56%	57%	55%	51%	45%
Rural	44%	43%	45%	49%	55%
Gender					
Male	49%	48%	52%	50%	50%
Female	51%	51%	48%	50%	50%
Partnership					
Single	14%	2%	3%	18%	78%
Partnered	79%	88%	94%	79%	20%
Other	7%	10%	3%	3%	2%
Education					
Elementary or below	23%	38%	17%	8%	3%
Junior high	29%	30%	36%	26%	21%
Senior high	23%	21%	20%	22%	32%
College	10%	5%	13%	17%	15%
Degree or above	15%	5%	14%	17%	29%
Occupation					
Non-manual	17%	9%	26%	34%	13%
Manual	16%	13%	28%	17%	8%
Own account	14%	8%	25%	22%	9%
Home-based	13%	13%	13%	15%	7%
Unemployed	8%	4%	4%	7%	9%
Other	34%	53%	4%	5%	53%
Income (logged)	10.951 (1.259)	10.684 (1.260)	10.822 (1.197)	11.022 (1.288)	10.756 (1.217)
Health	3.568 (0.915)	3.293 (0.943)	3.627 (0.838)	3.856 (0.788)	4.010 (0.768)
Region					
Four municipalities	13%	12%	13%	13%	12%
Western	17%	18%	17%	16%	17%
Central	23%	24%	22%	20%	23%
Eastern	40%	39%	41%	43%	42%
Northeastern	7%	7%	8%	7%	6%
Happiness (1–5)	3.694 (0.834)	3.712 (0.914)	3.699 (0.881)	3.702 (0.952)	3.633 (1.003)
Unweighted N	12,607	5853	2156	2311	1287

Source: CHFS 2015

Next, the empirical analysis takes into account a number of sociodemographic factors that have demonstrated noticeable effects on subjective well-being and public opinion on environmental issues in existing studies [6, 12, 15]. The ‘*hukou*’ variable distinguishes rural-to-urban migrants from urban-to-urban migrants. Respondents’ partnership statuses are classified into three categories, namely ‘single’, ‘partnered’ (i.e. married or cohabiting), and ‘other’ (i.e. divorced, widowed, or unanswered). A variable indicating respondent occupation was created based on the nature of an individual’s current full-time job or last full-time job before retirement. We classify cadres, managers, professionals, administrative personnel, clerical workers, and sales and service workers as the ‘non-manual’ class. Manual workers and farmers are assigned to the ‘manual’ category. Private entrepreneurs and self-employed respondents are assigned to the ‘own account’ category. Respondents who have never had a job or did not have a full-time job at the time of the survey interview are further classified into three categories. The ‘home-based’ group includes mainly housewives and those who choose to stay at home to look after dependent children or other family members. ‘Unemployed’ individuals are active job seekers who had failed to secure a full-time job in the six months prior to the survey interview. Full-time students, those with long-term illnesses, those unable to work, and all other respondents are assigned to the ‘other’ category. The effect of income is measured by logged annual household disposable income (*Chinese Yuan/Renminbi*). A merit of using logged income and nominal categories for occupational attainment is that they allow us to reduce multicollinearity among variables. To confirm this, we conduct an additional test to obtain variance inflation factors (VIF) for the independent variables, especially income, education, and occupation, which are likely to be confounding in a statistical model. The results confirm that most of the predictors display VIF that are lower than 2.0, with the exception of *hukou* (4.2).

Using a 5-point Likert scale (1 = ‘very bad’; 5 = ‘very good’), the CHFS asked its respondents to rate their overall health, and this is also included in our analysis. Finally, respondents’ host cities are classified into five geographical regions in line with the classification of the National Bureau of Statistics of China.

Importantly, the descriptive statistics in Table 1 show that the four generations of migrants vary significantly in terms of sociodemographic characteristics. Members of younger cohorts are more likely to be rural *hukou* holders. As far as socioeconomic status is concerned, younger cohorts appear to have higher educational qualifications, which is partly due to China’s higher education expansion in 1997. While older cohorts are more likely to be manual workers, younger cohorts are more likely to have non-manual occupations. A much larger proportion of Post-70s and Post-80s respondents belong to the ‘own account’ category than members of the other two cohorts. Not surprisingly, some inherent generational characteristics are also mirrored in Table 1. For example, members of the Post-90s cohort have a high propensity to be single, full-time students as compared to the others.

### Dependent variable

The CHFS does not include a module that systematically measure SWB using scaling strategies such as Affect Balance Scale), Positive and Negative Affect Scale and modified Differential Emotions Scale. Instead, it uses a primary scale of happiness measurement and asked the respondents the following question: ‘Overall, would you say you are happy?’ Response options used a 5-point Likert scale ranging from ‘not at all happy’ (coded as 1) to ‘very happy’ (coded as 5). This scale is also widely used in other major social surveys in China, such as the Chinese General Social Survey. The happiness scores of the migrant cohorts are summarized in Table 1.

### Analytical design

The empirical analysis in this paper consists of three parts and proceeds as follows. At the outset, we use latent class analysis (LCA) to explore potential forms of community environmental satisfaction in the study population. As a prominent branch of structural equation modelling, LCA addresses the complex pattern of association that appears among observations. The key logic of LCA is that the method is ‘respondent-centred’, as it allows us to classify the study population based on the proximity or distance of their scores on the variables. LCA models seek to assign individual respondents to one of a number of subgroups, i.e. classes with similar patterns. Using the classes captured in LCA as the dependent variable, we then establish a multinomial logistic regression model to examine the sociodemographic characteristics of different forms of community environmental satisfaction. The last analysis consists of a series of ordinal logistic regression models, which aim to capture the association between community environmental satisfaction and happiness among migrant cohorts in urban China.

## Results

### Exploring forms of community environmental satisfaction

The 2015 CFHS includes a set of questions covering six aspects of respondents’ level of satisfaction with their local communities. These aspects are ‘natural environment’, ‘social life (such as cultural and entertainment activities)’, ‘community safety’, ‘residential committee elections’, ‘road condition and infrastructure’, and ‘quality of local schools’. Response options include ‘very satisfied’, ‘quite satisfied’, ‘don’t know/no such item in the local community’, ‘not very satisfied’, and ‘not at all satisfied’; the options are dichotomized as ‘very satisfied’ or ‘quite satisfied’ versus other answers. Table 2 shows respondents’ rather low overall level of satisfaction with their local communities. The patterns also vary by different types of satisfaction items and by migrant cohort.

**Table 2:** Indicators for Community Environmental Satisfaction

Indicators	Weighted %				
	All	Pre-70s	Post-70s	Post-80s	Post-90s
Natural environment	18%	22%	18%	18%	17%
Social life (such as cultural and entertainment activities)	13%	9%	15%	14%	13%
Community safety	14%	13%	13%	16%	14%
Residential committee elections	10%	10%	10%	11%	9%
Road condition and infrastructure	20%	18%	25%	24%	17%
Quality of local schools	11%	14%	10%	9%	11%

Source: CHFS 2015

Results from the LCA are displayed in Tables 3 and 4. Table 3 indicates that a model postulating four latent classes fits the data adequately [30]. The log-likelihood and BIC suggest that going from two-class to five-class solutions offers little improvement in the fit of the model. The low AIC score for the four-class solution and the patterns that the entropy drops sharply from the four- to the five-class solution and that the LRT *p*-value is non-significant for the four-class solution suggest that five classes are not necessary. Table 4 shows the estimated size of the latent classes and the estimated probabilities, conditional on levels of satisfaction in each of the six manifest indicators. While conditional a probability greater than 0.3 tends to indicate considerable contribution to a latent class, we set the threshold to 0.5 in order to obtain more satisfying and rigorous results [30]. The largest latent class (Class 1), comprising nearly 50% of the sample, exhibits almost no satisfaction with any aspect. The 20% of the study population in Class 2 have high probabilities of being satisfied with community safety (64%) and residential committee elections (54%). By contrast, members of the smallest class (Class 3) have high probabilities of being satisfied with their natural environment (59%) and with road condition and infrastructure (55%). The fourth latent class, comprising 16% of the sample, presents a high probability of being satisfied with social life in the local community (71%). Interestingly, results from the LCA did not reveal any subgroup as being relatively satisfied with all six aspects of the local community. More importantly, the latent classes extracted from the LCA are able to mirror four different domains of migrants’ perception on local environment. Accordingly, we call Class 1 ‘dissatisfying local environment’. Classes 2, 3, and 4 are called ‘satisfying social environment’, ‘satisfying physical environment’, and ‘satisfying social life’, respectively. The LCA calculates conditional membership probabilities in each of the four latent classes for every respondent in the sample. Therefore, we assign each individual to a specific class according to the highest probability of conditional membership.

**Table 3:** Model fit of latent class measurement of social involvement

#classes	df	log-likelihood	AIC	BIC	entropy	LRT k-1 vs. k
1	178	− 1398.387	13,923.467	14,013.243	–	–
2	178	− 935.223	13,765.433	13,920.630	0.952	0.000
3	168	− 858.471	13,685.012	13,836.477	0.926	0.000
4	158	− 848.001	13,597.510	13,800.640	0.891	0.175
5	148	− 846.456	13,600.689	13,791.883	0.802	0.069

**Table 4:** Estimated size of the latent classes and the conditional probabilities of membership (*N* = 11,607)

	Latent class			
	1	2	3	4
*Relative size (%)*	0.499	0.202	0.138	0.161
Natural environment	0.012	0.059	0.592	0.211
Social life	0.000	0.007	0.002	0.708
Community safety	0.000	0.642	0.091	0.142
Residential committee elections	0.000	0.544	0.073	0.044
Road condition and infrastructure	0.000	0.112	0.555	0.002
Quality of local schools	0.021	0.221	0.036	0.002

*Note:* Shaded cells are conditional probabilities greater than 0.500

### Sociodemographic determinants of community environmental satisfaction

To examine the effects of sociodemographic factors on community environmental satisfaction, we constructed a multinomial logistic regression model that uses the four latent classes as the dependent variable. Model results are presented in Table 5, in which ‘dissatisfying environment’ is used as the omitted category.

**Table 5:** Sociodemographic characteristics of the latent classes

	Satisfying social environment	Satisfying physical environment	Satisfying social life
Cohorts (*Pre-70s*)			
Post-70s	−0.023***	− 0.203***	− 0.169***
Post-80s	− 0.259***	− 0.526***	− 0.313***
Post-90s	− 0.385***	− 0.144***	− 0.096***
Gender (*Male*)			
Female	−0.097***	− 0.013***	− 0.177***
Partnership (*Single*)			
Partnered	− 0.334***	− 0.146***	− 0.139***
Other	− 0.167***	− 0.238***	− 0.018***
*Hukou* (*Non-rural*)			
Rural	− 0.380***	− 0.420***	− 0.114***
Education (*elementary or below*)			
Junior high	−0.045***	− 0.122***	− 0.086***
Senior high	−0.099***	− 0.133***	− 0.225***
College	− 0.176***	− 0.629***	− 0.320***
Degree or above	− 0.307***	− 0.655***	− 0.684***
Occupation (*Manual*)			
Non-manual	− 0.442***	− 0.321***	− 0.141***
Own account	− 0.196***	− 0.054***	− 0.207***
Home-based	− 0.367***	− 0.256***	− 0.490***
Unemployed	− 0.298***	− 0.098***	− 0.496***
Other	− 0.055***	− 0.178***	− 0.207***
Logged income	− 0.007***	− 0.133***	− 0.077***
Health	− 0.025***	− 0.100***	− 0.086***
Region (*Four municipalities*)			
Western	− 0.238***	− 0.173***	− 0.134***
Central	− 0.227***	− 0.019***	− 0.017***
Eastern	− 0.051***	− 0.499***	− 0.202***
Northeastern	− 0.107***	− 0.230***	− 0.022***
Constant	−1.979***	−2.665***	−2.573***
N	12,607		
Log-likelihood	− 1287.453		
Pseudo R^2^	0.023		

Note: *p < 0.05 **p < 0.01 ***p < 0.001

In regard to the effects of cohorts, we can see that the Post-80s and Post-90s cohorts are more likely than the Post-70s cohort to be in the ‘satisfying social environment’ rather than the ‘dissatisfying environment’ category, while there is a similar contrast between the Post-70s and the Post-80s cohorts in the ‘satisfying physical environment’ column. Moreover, the effect of *hukou* status is also significant. As compared to urban-to-urban migrants, rural-to-urban migrants appear to be less likely to be satisfied with the environment of the local community in their host cities, not least in terms of social environment and physical environment. It is largely resulted from the fact that rural-to-urban migrants are more likely to live in worse environment, such as informal rental housing, by comparing to their counterparts [22, 31, 32]. Turning to the coefficients of education, we are able to discern a clear gradient associated with the effect sizes of different levels of educational attainment. Migrants with college or higher qualifications appear to be more likely than others to be satisfied with the physical environment of their local community, whereas they exhibit a low likelihood to express satisfaction with their social environments and social lives. To some extent it reflects that migrants with higher educational attainment are more likely to live in a better living environment [32]; while it also suggests that they tend to have more demands for non-physical conditions of the local community as social environment and social life. Similar contrasts are discernible between respondents in non-manual and manual occupations. Income and health present strong and positive effects in the column for satisfying physical environment. As far as regional variations are concerned, Table 5 shows that migrants in China’s western and central regions have higher propensities to belong to the latent class for satisfying social environment relative to that for dissatisfying environment, while the effect of living in the eastern region takes the opposite form in the column for satisfying physical environment.

### Migrant generations, community environmental satisfaction and happiness

Finally, we constructed a series of ordinal logistic regression models to examine the association between different forms of community environmental satisfaction and happiness across the four migrant cohorts (Table 6). The first model includes the whole sample, while each of the four migrant cohorts is then modelled separately. Looking at Model 1, we find that, when other variables are conditioned, satisfying physical environment and social life are significantly associated with greater happiness among migrants in urban China. In regard to cohort differences, the positive effect of a satisfying physical environment is observable in all four migrant cohorts. It is also worth noting that its effect size tends to increase in younger cohorts. Interestingly, the patterns for a satisfying social life take exactly the opposite form: the positive association between a satisfying social life and happiness is considerably more observable in the sample’s older cohorts. A satisfying social environment exhibits positive effects on happiness among the Pre-70s and Post-90s cohorts, and these effects are relatively weaker among the other two cohorts.

**Table 6:** Ordinal logistic regression coefficients on happiness

	Model				
	All	Pre-70s	Post-70s	Post-80s	Post-90s
Environmental satisfaction					
Satisfying social environment	−0.242***	− 0.285***	− 0.117***	−0.111***	− 0.486***
Satisfying physical environment	−0.500***	− 0.393***	−0.427***	− 0.563***	−0.654***
Satisfying social life	−0.749***	−0.985***	− 0.894***	−0.612***	− 0.264***
Gender (*Male*)					
Female	−0.557***	−0.026***	− 0.139***	−0.021***	− 0.094***
Partnership (*Single*)					
Partnered	−0.680***	−0.634***	− 0.731***	−0.703***	− 0.756***
Other	−0.182***	− 0.031***	−0.237***	− 0.169***	−0.229***
*Hukou* (*Non-rural*)					
Rural	−0.199***	−0.220***	− 0.173***	−0.265***	− 0.192***
Education (*elementary or below*)					
Junior high	−0.162***	−0.173***	− 0.320***	−0.300***	− 0.627***
Senior high	−0.011***	− 0.147***	−0.551***	− 0.016***	−0.201***
College	−0.047***	−0.338***	− 0.642***	−0.464***	− 0.274***
Degree or above	−0.010***	− 0.154***	−0.962***	− 0.196***	−0.614***
Occupation (*Manual*)					
Non-manual	−0.066***	−0.212***	− 0.171***	−0.169***	− 0.702***
Own account	−0.188***	− 0.213***	−0.240***	− 0.170***	−0.039***
Home-based	−0.336***	−0.369***	− 0.378***	−0.180***	− 0.593***
Unemployed	−0.123***	− 0.270***	−0.657***	− 0.541***	−0.559***
Other	−0.138***	−0.004***	− 0.197***	−0.011***	− 0.193***
Logged income	−0.102***	− 0.071***	−0.117***	− 0.096***	−0.164***
Health	−0.509***	−0.492***	− 0.749***	−0.354***	− 0.550***
Region (*Four municipalities*)					
Western	−0.133***	−0.097***	− 0.401***	−0.319***	− 0.160***
Central	−0.016***	− 0.047***	−0.436***	− 0.029***	−0.307***
Eastern	−0.345***	−0.297***	− 0.481***	−0.302***	− 0.460***
Northeastern	−0.290***	− 0.010***	−0.619***	− 0.579***	−0.112***
N	12,607	5853	2156	2311	1287
Log-likelihood	− 1626.390	− 1088.091	− 991.321	− 1071.362	−891.715
Pseudo R^2^	0.056	0.048	0.096	0.071	0.051

Note: *p < 0.05 **p < 0.01 ***p < 0.001

Turning to the effects of other independent variables, we can see that individuals who are partnered, wealthier, and healthier tend to report a higher level of happiness. Rural-to-urban migrants also display gaps in happiness with urban-to-urban migrants in most models, with the exception of members of the Post-90s cohort. Overall, migrants in the four municipalities appear to be less happy than their counterparts in other Chinese cities; this is particularly evident among the Post-70s cohort.

## Discussion

An extensive literature has investigated environmental satisfaction at the neighbourhood and community levels; however, little information has been produced on the implications of varying forms of community environmental satisfaction for individuals’ SWB [33]. To extend the literature on both community satisfaction and SWB, this article first explores forms of community environmental satisfaction and then addresses their impacts on migrants’ SWB. Our technique was to use latent class analysis to identify four different domains of migrants’ perception of local environment, namely ‘dissatisfying local environment’, ‘satisfying social environment’, ‘satisfying physical environment’, and ‘satisfying social life’. Notwithstanding an overall low level of community satisfaction, the results of the LCA were able to detect social environment, physical environment, and social life as three distinctive components of community environmental satisfaction among migrants in urban China.

Further analysis shows that different forms of community environmental satisfaction tend to reflect differences in social hierarchy and demography. Income, educational qualifications and occupational attainment are all significantly and positively associated with greater levels of satisfaction with one’s physical environment and with lower levels of satisfaction with one’s social environment and social life. To a certain extent, this discovery suggests that, while affluent migrants are able to invest more economic capital to improve their physical living environments, their demands for such non-physical conditions of the local community as social environment and social life increase simultaneously. This finding is consistent with existing studies positing that individuals with higher incomes and better health often live in better physical environments [34, 35]. Regional differences are also observable in the analysis. Although our analysis did not use regions as sub-samples for the analysis due to restriction of the sample size, it is plausible that the socioeconomic characteristics of environmental satisfaction may well be distinct between different Chinese regions, considering that migration patterns vary significantly across cities in China [1, 4].

One of the most striking findings in our analysis is the significant contribution of community-based social environment and social life to migrants’ happiness. Importantly, this finding suggests that migrants’ happiness is largely determined by both the ‘soft’, non-physical environment and the ‘hard’, physical environment of the local community. It lends some support to Li and Liu’s recent finding that, as compared to housing conditions, neighbourhood social environment is a much stronger predictor of mental health among the migrant population in urban China [36]. In particular, the significant effect size of having a satisfying social life coincides with the observation in the previous literature that neighbourhood attachment, social support, and social connectedness are key ingredients of SWB [37–39]. Although many Chinese citizens have migrated to the city for economic reasons, our analysis is able to reveal that the ‘social’ constituencies of local community, which are mirrored essentially in an inclusive environment underpinned by formal community-based communal participation and informal in-person contacts, are likely to play an equally important role in shaping urban migrants’ happiness. While our finding tends to confirm the observation that social exclusion erodes subjective well-being among migrants in urban China, it further points towards the positive implications that an inclusive community may present to its migrant members [22, 36].

Nevertheless, the analysis in this study also finds that cohort differences play an important role in shaping the association between community life satisfaction and happiness. Given the substantive changes that have taken place in urban China during the past four decades, there is mounting evidence of cohort and generational differences among migrants. For instance, migrants of different generations are distinctive in social identity [25], social networks [26], urban settlement intentions [40], and urban experiences [4]. Our results suggest that cohort differences do exist and, furthermore, that they appear to mirror differences in sources of happiness. Interestingly, the positive effect of a satisfying physical environment on happiness tends to increase in younger cohorts, while the positive effect of a satisfying social life on happiness is more observable in the older cohorts. This reflects greater demands with respect to material enjoyment among migrants in younger cohorts [4] and less reliance on social life [26]. It is worth noting that migrants’ sociodemographic characteristics are significantly associated with their levels of happiness. For the sample as a whole and for samples of different cohorts, partnership, income and health are all significantly related to migrants’ happiness. In addition, spatial differences are discernible in that migrants from the four municipalities are shown to be less happy than those in other regions.

In sum, the findings of this study demonstrate that different forms of community satisfaction, in particular a satisfying physical environment and social life, play important roles in explaining individuals’ happiness. As we know, the majority of migrants to Chinese cities live in marginal areas such as urban villages and dilapidated neighbourhoods [41]. In order to improve migrants’ SWB, policy makers should pay more attention to migrants’ physical living environments. Additionally, existing studies have revealed that migrants usually devote extremely long hours to their work in order to secure better incomes, spending little time on leisure and social life [42, 43]. According to our study, this is detrimental to migrants’ SWB. As such, regulation of the employment market and improvements in migrants’ salaries without overtime work is another way in which migrants’ SWB could be improved.

## Conclusions

Improving migrants’ well-being has become a major concern for China’s state government. Previous studies on SWB have focused primarily on the impacts of objectively measured community environment and consider migrants as a homogeneous group. This study extends the literature by exploring the impacts of perceived community environment on migrants’ happiness and incorporating cohort differences in the analysis. Our findings suggest that improving migrants’ physical living environments and social lives is more important than social environment to their happiness, which in a way mirrors migrants’ current status, typified by uncomfortable living environments and deficient social lives. Thus, we suggest that policy reforms prioritize improvements in migrants’ physical living environments and regulate the employment market. Moreover, there exist prominent cohort differences, and these tend to reflect differences in the sources of happiness. This finding confirms that migrants in Chinese cities are no longer a homogeneous group; it is important to enhance migrants’ levels of happiness by considering the unique characteristics of different cohorts and generations. Future research should include the objective aspects of community environment and compare their impacts and mechanisms on migrants’ SWB. It would also be interesting to compare migrants with urban natives.

## Abbreviations

CHFS: China household finance survey
LCA: Latent class analysis
SWB: Subjective well-being

## References

[1] Wu W (2002). Migrant housing in urban China: choices and constraints. Urban Aff Rev.

[2] Meng X, Zhang J (2001). The two-tier labor market in urban China: occupational segregation and wage differentials between urban residents and rural migrants in Shanghai. J Comp Econ.

[3] Zhang L, Ma LJC, Wu F (2005). Migrant Enclaves and Impacts of Rdevelopment Policy in Chinese Cities, in Restructuring the Chinese City: Changing Society, Economy and Space.

[4] He S, Wang K (2016). China’s New Generation Migrant Workers’ Urban Experience and Well-Being.

[5] Liu Y (2017). The effect of neighbourhood social ties on migrants' subjective wellbeing in Chinese cities. Habitat International.

[6] Sun S (2016). Subjective well-being and its association with subjective health status, age, sex, region, and socio-economic characteristics in a Chinese population study. J Happiness Stud.

[7] Cai S, Wang J. Less advantaged, more optimistic? Subjective well-being among rural, migrant and urban populations in contemporary China. China Econ Rev. 2018.

[8] Cheng Z, Wang H, Smyth R (2013). Happiness and job satisfaction in urban China: a comparative study of two generations of migrants and urban locals. Urban Stud.

[9] Diener E (1984). Subjective well-being. Psychol Bull.

[10] Knight J, Gunatilaka R (2010). Great expectations? The subjective well-being of rural–urban migrants in China. World Dev.

[11] De Neve JE, Diener E, Tay L, Xuereb C The Objective Benefits of Subjective Well-Being (August 6, 2013). In Helliwell, J., Layard, R., & Sachs, J., edn. World Happiness Report 2013. New York: UN Sustainable Development Solutions Network.

[12] Anderson C (2012). The local-ladder effect: social status and subjective well-being. Psychol Sci.

[13] Diener E, Biswas-Diener R (2002). Will money increase subjective well-being?. Soc Indic Res.

[14] Cummins RA (2000). Personal income and subjective well-being: a review. J Happiness Stud.

[15] Diener E, Oishi S (2000). Money and happiness: Income and subjective well-being across nations.

[16] Dong H, Qin B (2017). Exploring the link between neighborhood environment and mental wellbeing: a case study in Beijing, China. Landsc Urban Plan.

[17] Liu Y, Dijst M, Geertman S (2016). The subjective well-being of older adults in Shanghai: the role of residential environment and individual resources. Urban Stud.

[18] Liu Y (2017). The subjective wellbeing of migrants in Guangzhou, China: the impacts of the social and physical environment. Cities.

[19] Cao X (2016). How does neighborhood design affect life satisfaction?. Evidence from Twin Cities Travel Behaviour and Society.

[20] Institute of Medicine (2003). The future of the public’s health in the 21st century.

[21] McNeill LH, Kreuter MW, Subramanian SV (2006). Social environment and physical activity: a review of concepts and evidence. Soc Sci Med.

[22] Li J, Rose N (2017). Urban social exclusion and mental health of China's rural-urban migrants – a review and call for research. Health Place.

[23] Gidlöf-Gunnarsson A, Öhrström E (2007). Noise and well-being in urban residential environments: the potential role of perceived availability to nearby green areas. Landsc Urban Plan.

[24] Logan J, Collver A (1983). Residents' perceptions of suburban community differences. Am Sociol Rev.

[25] Wang C (2001). Social identity of the new generation of rural hobo and merger of urban and rural. Sociol Res.

[26] Liu Y, Li Z, Breitung W (2012). The social networks of new-generation migrants in China’s urbanized villages: a case study of Guangzhou. Habitat International.

[27] Yang J. The social integration difficulty for China's post-90s migrants. Chinese Social Sciences Today. 2016;5(19). http://www.cssn.cn/zx/201511/t20151118_2670570.shtml.

[28] Egri CP, Ralston DA (2004). Generation cohorts and personal values: a comparison of China and the United States. Organ Sci.

[29] Hu, Y., Post-1980s generation wields power, in Asia Weekly. 2017, Chinadaily Asia.

[30] Mccutcheon AL (2002). Basic concepts and procedures in single- and multiple-group latent class analysis. Applied latent class analysis.

[31] Huang Y, Tao R (2014). Housing migrants in Chinese cities: current status and policy design. Environment & Planning C.

[32] Tao L, Wong FKW, Hui ECM (2014). Residential satisfaction of migrant workers in China: a case study of Shenzhen. Habitat International.

[33] Theodori GL (2001). Examining the effects of community satisfaction and attachment on individual well-being. Rural Sociol.

[34] Gangadharan L, Valenzuela MR (2001). Interrelationships between income, health and the environment: extending the environmental Kuznets curve hypothesis. Ecol Econ.

[35] Scopelliti M, et al. Staying in touch with nature and well-being in different income groups: the experience of urban parks in Bogotá. Landsc Urban Plan. 2016;148:139–48.

[36] Li J, Liu Z (2018). Housing stress and mental health of migrant populations in urban China. Cities.

[37] Huang Y. The dynamics of online activities and its impact on well-being in urban China. Soc Sci. 2018;7.

[38] Helliwell JF (2010). Well-being, social capital and public policy: What's new?. Economic Journal.

[39] Wang R (2018). The relationship between urbanization and depression in China: the mediating role of neighborhood social capital. Int J Equity Health.

[40] Yue Z (2010). Floating choices: a generational perspective on intentions of rural–urban migrants in China. Environment and Planning A: Economy and Space.

[41] Liu Y (2010). Urban villages under China's rapid urbanization: unregulated assets and transitional neighbourhoods. Habitat International.

[42] Lin S, Gaubatz P (2017). Socio-spatial segregation in China and migrants’ everyday life experiences: the case of Wenzhou. Urban Geogr.

[43] Huang Y. Social capital and social trust in urban China. Chin J Sociol. 2018;4(4):481–505.

