# Correction to: Community environmental satisfaction: its forms and impact on migrants’ happiness in urban China

**DOI:** 10.1186/s12955-019-1213-y

**Published:** 2019-10-24

**Authors:** Sainan Lin, Yinxuan Huang

**Affiliations:** 1 School of Urban Design, Wuhan University, Wuhan, China; 2 Manchester Urban Institute, School of Environment, Education, and Development, The University of Manchester, 1.34 Humanities Bridgeford Street Building, Oxford Road, Manchester, M13 9PL UK

**Correction to: Health Qual Life Outcomes (2018) 16:236.**

**https://doi.org/10.1186/s12955-018-1061-1**

The original article [1] contains errors in the presentation of Table 5 & Table 6 whereby values are displayed in an incorrect format leading to potential misinterpretation. The correctly presented versions of Table 5 & Table 6 can be viewed ahead in this Correction article.

**Table 5:** Sociodemographic characteristics of the latent classes

	Satisfying social environment	Satisfying physical environment	Satisfying social life
Cohorts (*Pre-70s*)			
Post-70s	-0.023***	-0.203***	-0.169***
Post-80s	-0.023	0.203	-0.169
Post-90s	0.259*	0.526***	-0.313
Gender (*Male*)	0.385*	0.144	0.096
Female			
Partnership (*Single*)	-0.097	0.013	0.177
Partnered			
Other	0.334	0.146	0.139
*Hukou* (*Non-rural*)	0.167	0.238	-0.018
Rural			
Education (*elementary or below*)	-0.380*	-0.420**	-0.114
Junior high			
Senior high	-0.045	0.122	-0.086
College	-0.099	0.133	-0.225
Degree or above	-0.176	0.629***	-0.320*
Occupation (*Manual*)	-0.307*	0.655***	-0.684**
Non-manual			
Own account	-0.442**	0.321*	-0.141
Home-based	0.196	-0.054	0.207
Unemployed	0.367*	0.256	0.490**
Other	0.298	0.098	0.496**
Logged income	0.055	0.178	0.207
Health	0.007	0.133**	0.077
Region (*Four municipalities*)	0.025	0.100*	0.086
Western			
Central	0.238*	0.173	-0.134
Eastern	0.227*	0.019	-0.017
Northeastern	0.051	-0.499***	-0.202
Constant	0.107	0.230	-0.022
N		12,607	
Log-likelihood		-1287.453	
Pseudo R^2^		0.023	

Note: *p<0.05 **p<0.01 ***p<0.001

**Table 6:** Ordinal logistic regression coefficients on happiness

	Model				
	All	Pre-70s	Post-70s	Post-80s	Post-90s
Environmental satisfaction					
Satisfying social environment	0.242	0.285*	0.117	0.111	0.486*
Satisfying physical environment	0.500**	0.393*	0.427*	0.563*	0.654***
Satisfying social life	0.749***	0.985***	0.894***	0.612**	0.264
Gender (*Male*)					
Female	0.557	0.026	0.139	0.021	0.094
Partnership (*Single*)					
Partnered	0.680***	0.634***	0.731***	-0.703***	-0.756***
Other	0.182	0.031	-0.237	-0.169	-0.229
*Hukou* (*Non-rural*)					
Rural	-0.199**	-0.220*	-0.173	-0.265*	0.192
Education (*elementary or below*)					
Junior high	-0.162	-0.173	0.320	-0.300	-0.627
Senior high	-0.011	-0.147	0.551	0.016	0.201
College	0.047	0.338	0.642*	-0.464	0.274
Degree or above	0.010	-0.154	0.962**	-0.196	0.614
Occupation (*Manual*)					
Non-manual	-0.066	0.212	-0.171	-0.169	-0.702
Own account	0.188	0.213	0.240	0.170	0.039
Home-based	0.336	0.369	0.378	0.180	0.593
Unemployed	-0.123	0.270	-0.657	-0.541	-0.559
Other	0.138	0.004	-0.197	0.011	-0.193
Logged income	0.102***	0.071*	0.117*	0.096*	0.164**
Health	0.509***	0.492***	0.749***	0.354***	0.550***
Region (*Four municipalities*)					
Western	0.133	0.098	0.401*	0.319	-0.160
Central	0.016	0.047	0.436*	-0.029	-0.307
Eastern	0.345***	0.297**	0.481**	0.302	0.460
Northeastern	0.290***	0.010	0.619*	0.579*	0.112
N	12,607	5,853	2,156	2,311	1,287
Log-likelihood	-1626.390	-1088.091	-991.321	-1071.362	-891.715
Pseudo R^2^	0.056	0.048	0.096	0.071	0.051

Note: *p<0.05 **p<0.01 ***p<0.001
